# Global trends and hotspots in artificial intelligence for high myopia: a bibliometric analysis

**DOI:** 10.3389/fmed.2025.1567440

**Published:** 2025-05-09

**Authors:** Xuze Wang, Ailixiati Wumaier, Jun Wang, Dejuan Song, Yiting Cai, Jin Han, Wei Han, Zhi Fang

**Affiliations:** Zhejiang Provincial Key Laboratory of Ophthalmology, Zhejiang Provincial Clinical Research Center for Eye Diseases, School of Medicine, Eye Center of Second Affiliated Hospital, Zhejiang Provincial Engineering Institute on Eye Diseases, Zhejiang University, Hangzhou, China

**Keywords:** high myopia, artificial intelligence, global research, bibliometric analysis, data visualization

## Abstract

**Purpose:**

This study aims to conduct a bibliometric analysis of global publications on the application of artificial intelligence (AI) in high myopia (HM).

**Methods:**

We retrieved publications on AI in HM from the Web of Science Core Collection (WoSCC) database, MEDLINE and Chinese Science Citation Database (CSCD) with data up to 2024. The analysis focused on publication and citation trends, identifying key articles, influential countries, institutions, authors, and journals. Additionally, we explored research domains and emerging keywords.

**Results:**

A total of 167 relevant publications were included. The first AI-related paper on HM was published in 2017, with a significant surge in 2021, followed by a consistent increase in publication and citation counts over the next 3 years. China emerged as the most productive country, with the most extensive international collaboration. East Asian authors dominated the top 10 most influential authors. Yang, Weihua and Investigative Ophthalmology & Visual Science (IOVS) contributed the most publications among authors and institutions, respectively. Keyword analysis revealed that retinal imaging-related terms remained a consistent research focus, while newly emerging keywords included “automated detection” and “childhood.”

**Conclusion:**

Recent advancements in AI applications for HM have been significant and are expected to continue. Future research will likely focus on multimodal imaging and improving algorithm accessibility. Our findings offered the first comprehensive overview of global research on AI in HM, thus providing valuable insights for researchers to understand the current status and future trends in this field.

## Introduction

Myopia is one of the significant global healthcare challenges, with high myopia (HM) being a particular concern (1–3). HM is defined as a condition in which the spherical equivalent refractive error is ≤ −6.00 D when ocular accommodation is relaxed (4). By 2050, HM is expected to affect 9.8% of the global population (5). The axial elongation associated with HM increases the risk of structural changes in the posterior segment of the eye, including posterior staphyloma, myopic maculopathy, and HM-related optic neuropathy (3, 4, 6). These changes may lead to a decline in best-corrected visual acuity (7). Additionally, HM serves as a foundational ocular condition for many other eye diseases, complicating their diagnosis and treatment (6–9). Therefore, research into HM is of significant importance.

Artificial intelligence (AI) has gained significant global attention in recent years (10). AI offers unique advantages in medical imaging analysis by enabling systems to extract valuable information from digital images for advanced analysis (11, 12). Since ocular imaging is fundamental to ophthalmology, AI holds enormous potential in this field (11, 13–15). AI-based large language models (LLMs) also contribute to the equitable distribution of ophthalmic healthcare resources and community monitoring of chronic eye diseases (16). HM research is especially well-suited for AI, with applications like fundus image analysis for HM-related retinopathy and deep learning models (DLMs) predicting HM progression and complications (11–13, 16). In fact, numerous studies have already been published in this area.

Bibliometric analysis is a statistical method that quantitatively assesses research achievements and identifies hotspots by evaluating the research status of countries, institutions, authors, and journals (17, 18). With a history spanning over a century, bibliometric analysis has been widely applied in medicine to explore its development and emerging trends (19, 20). While bibliometric studies on myopia and HM have been conducted (21, 22), there is a lack of bibliometric analysis on AI in HM. This gap has become even more pronounced as AI-related HM research has surged in recent years, making it challenging to identify key areas and current trends (23–25). This highlights the urgent need for a bibliometric analysis of AI-related HM research. Therefore, our study aims to address this challenge by retrieving relevant papers from the Web of Science Core Collection (WoSCC), MEDLINE and Chinese Science Citation Database (CSCD), providing an overview of global AI-related HM research, identifying key themes, and predicting future trends. These insights are valuable for ophthalmic clinicians and researchers.

## Methods

### Data collection

The WoSCC is known for its rigorous selection criteria and high-quality coverage across multiple disciplines, including journals, conference papers, and patents (26, 27). MEDLINE, as a core component of PubMed, offers over 31 million biomedical references and is widely recognized in life sciences research (28). The CSCD provides structured and accurate data on Chinese academic output (27). All three databases are considered ideal for bibliometric analysis due to their authority, broad coverage, and data quality. To search for relevant data, we combined at least one keyword related to HM and at least one keyword related to AI to form the query. The detailed query formulation was as follows: TS = (“AI” OR “artificial intelligence” OR “intelligent” OR “data learning” OR “robotic*” OR “computer vision” OR “machine learning” OR “deep learning” OR “deep network*” OR “neural learning” OR “algorithm” OR “neural network*” OR “expert* system*” OR “large language model*” OR “LLM” OR “multimodal model*” OR “multimodal learning” OR “transformer model*” OR “AI* classification” OR “image segmentation” OR “domain adaptation” OR “model generalization” OR “feature extraction” OR “object detection” OR “model interpretability” OR “transfer learning”) AND TS = (“high myopia” OR “pathologic myopia” OR “degenerative myopia” OR “progressive myopia” OR “myopic maculopathy” OR “myopic choroidopathy” OR “myopic degeneration” OR “highly myopic eyes” OR “high myopic patients” OR “high degree myopia” OR “severe myopia”). The search timeframe was extended until December 31, 2024, and the document types were limited to articles and proceedings papers. The final search was conducted on April 14, 2025. A total of 356 documents were identified for further screening.

### Data screening

To exclude irrelevant documents from the retrieved set, we established the following inclusion criteria: (i) involvement of AI technology, including deep learning, machine learning, and LLM, etc.; (ii) the focus should be on HM or its related complications, diagnoses, and treatments, etc. If the study included multiple diseases, HM condition should be the primary focus. After a careful review of their titles and abstracts by two ophthalmologists, Wang Xuze and Fang Zhi, 167 documents were included for the bibliometric analysis.

### Bibliometric analysis

All the data were extracted from the three aforementioned databases, including metrics of publication numbers, countries and regions, authors, citations, self-citations, and H-indexes. The H-index serves as a reference metric, reflecting the impact of a researcher, country, institution, or journal on the development of a specific scientific field (29). Descriptive indices were extracted from databases, and the co-occurrence network was constructed using VOSviewer (software, version 1.6.20) (30). R (programming language, version 4.4.2) and its Biblioshiny tool (package, version 4.1.2) were employed to create word-cloud maps and analyze word trends (31). CiteSpace (software, version 6.3.1) was used to generate keyword burst detection maps (32).

## Results

### Analysis of publications and citations

**Figure 1:**
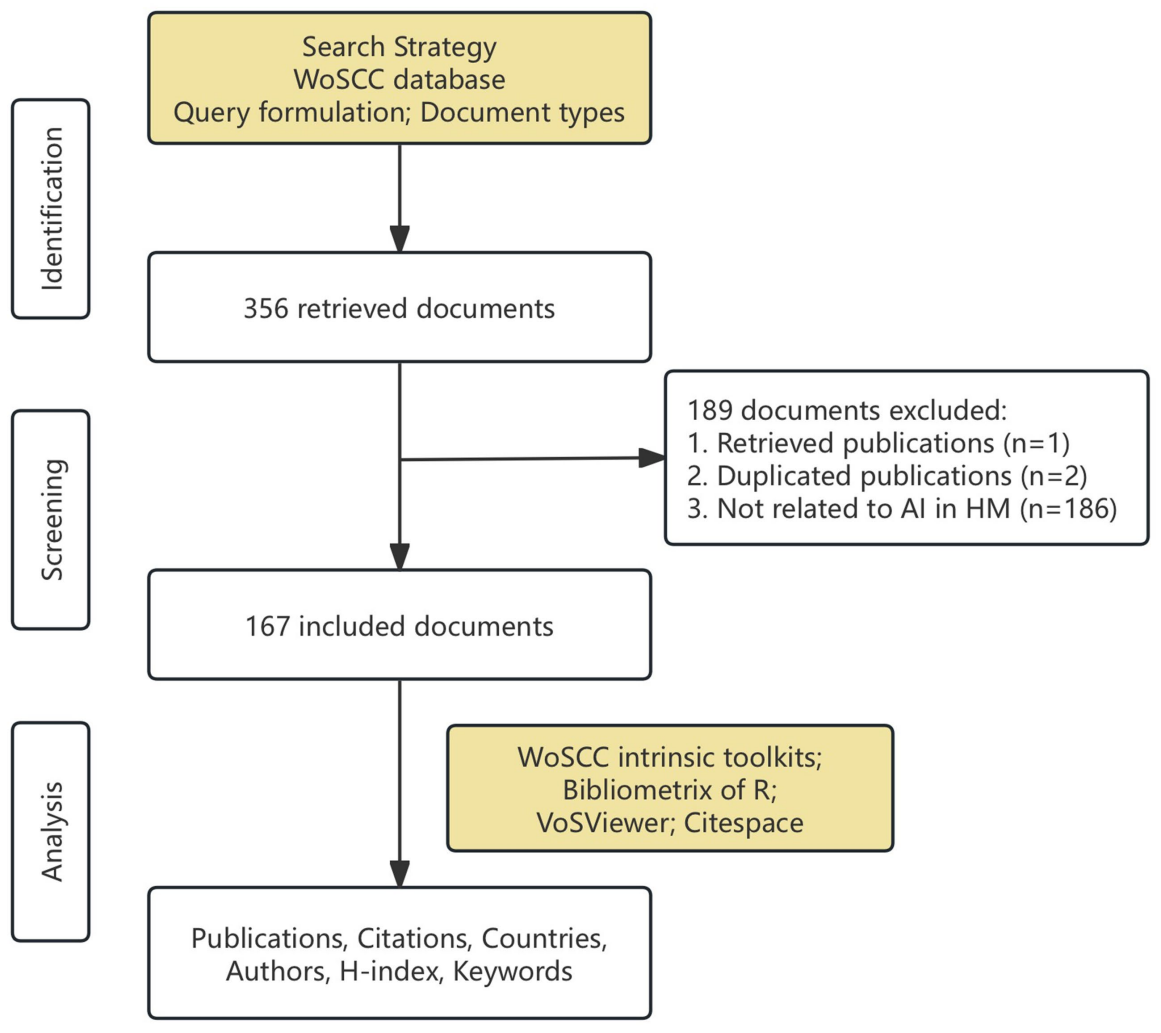
Detailed flowchart of this study.

**Figure 2:**
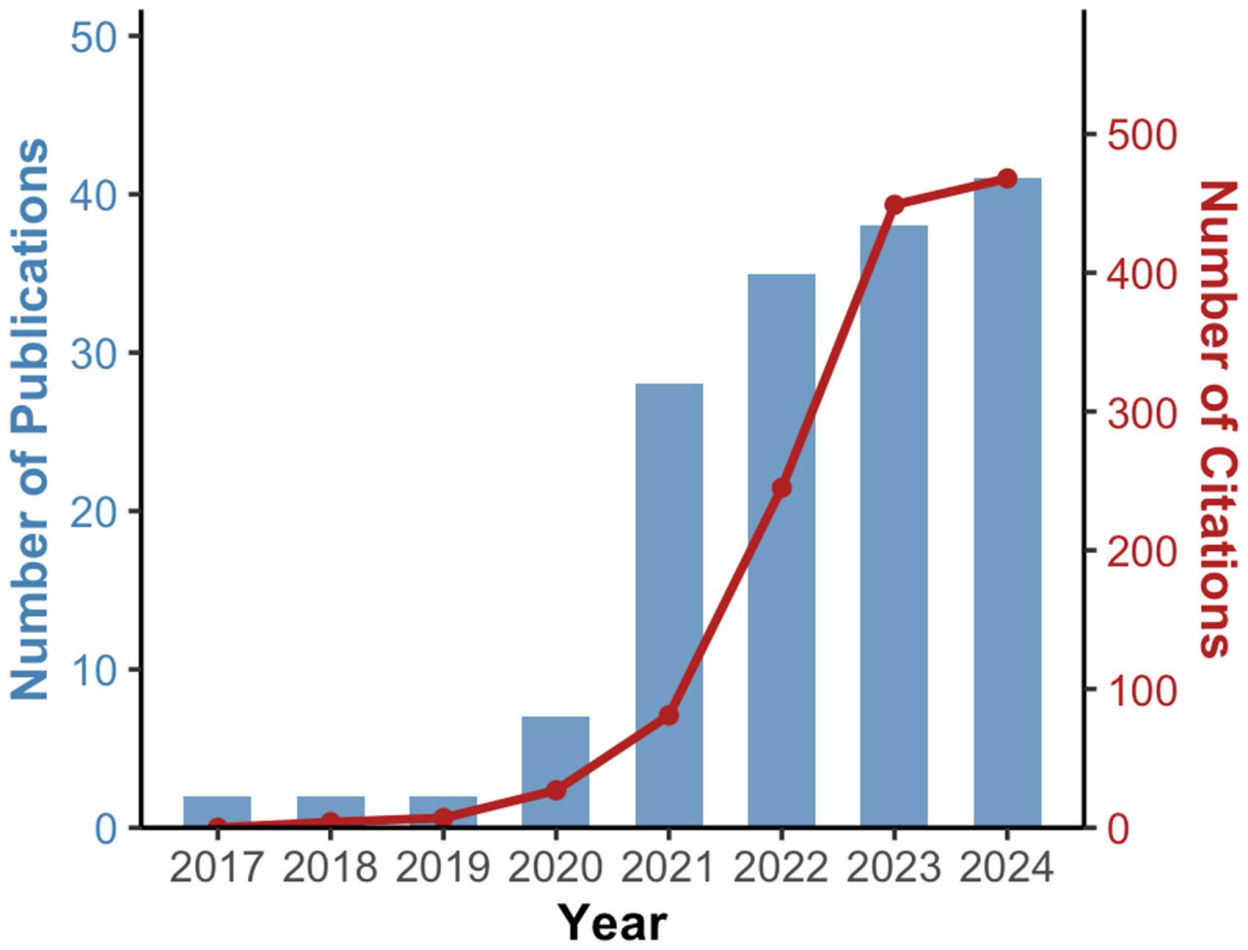
Trends in the number of AI-related publications and citations in HM.

Based on the search strategy and inclusion criteria, 167 documents were included (Figure 1), consisting of 144 articles and 23 conference proceedings, published between 2017 and 2024. We conducted a second review of 32 articles published before 2017 from the results retrieved using the above search strategy. They were excluded since HM was not the primary focus or lacking of AI. Figure 2 illustrated the annual trends in publications and citations related to AI in HM. Before 2020, research on AI in HM was limited, but there was a significant increase in publications since then, particularly in the past 3 years, with annual publications consistently exceeding 30. The total number of citations reached 1,402, with an average of 8.40 citations per article and an H-index of 20. Polynomial regression analysis was performed to model the publication and citation trends. The fitting curves, y = 0.3393×2-1363.9x + 1E+06 (*R*^2^ = 0.9248) and y = 15.964×2-64431x + 7E+07 (*R*^2^ = 0.9738), represented the publication and citation numbers over time, respectively. Both trends were similar, indicating that AI in the HM field attracted widespread attention from researchers in the past 5 years, with rapid development now reaching a stable phase.

Table 1 highlighted the 10 most-cited papers in the field. The most impactful study, conducted by Yizhi Liu and his team at Sun Yat-sen University, was published in 2018 in PLOS Medicine (33). This paper detailed the application of big data and machine learning technology to develop an algorithm capable of predicting the onset and prognosis of HM among Chinese school-aged children. The remaining top 10 most-cited papers primarily focused on the retinal conditions associated with HM and related AI applications.

**Table 1:** Top 10 papers with the most citations relevant to AI applications in HM.

Title	Corresponding authors	Journal	Publication year	Annual citations	Total citations
Prediction of myopia development among Chinese school-aged children using refraction data from electronic medical records: A retrospective, multicenter machine learning study	Liu, YZ	PLOS MEDICINE	2018/11	14.5	113
Retinal photograph-based deep learning algorithms for myopia and a blockchain platform to facilitate artificial intelligence medical research: a retrospective multicohort study	Ting, DSW	LANCET DIGITAL HEALTH	2021/5	20	91
Hybrid Intelligence-Driven Medical Image Recognition for Remote Patient Diagnosis in Internet of Medical Things	Yu, KP	IEEE JOURNAL OF BIOMEDICAL AND HEALTH INFORMATICS	2022/12	23.5	91
Association Between Optic Nerve Head Deformation and Retinal Microvasculature in High Myopia	Park, SW	AMERICAN JOURNAL OF OPHTHALMOLOGY	2018/4	7.75	59
Deep Learning Approach for Automated Detection of Myopic Maculopathy and Pathologic Myopia in Fundus Images	Ohno-Matsui, K	OPHTHALMOLOGY RETINA	2021/12	10.6	50
Development and validation of a deep learning system to screen vision-threatening conditions in high myopia using optical coherence tomography images	Lin, HT	BRITISH JOURNAL OF OPHTHALMOLOGY	2022/5	11.25	45
Pathological myopia classification with simultaneous lesion segmentation using deep learning	De Boever, P	COMPUTER METHODS AND PROGRAMS IN BIOMEDICINE	2021/2	9	41
Accuracy of a deep convolutional neural network in the detection of myopic macular diseases using swept-source optical coherence tomography	Mitamura, Y	PLOS ONE	2020/4	7.6	35
Accuracy of Artificial Intelligence Formulas and Axial Length Adjustments for Highly Myopic Eyes	Wu, MX	AMERICAN JOURNAL OF OPHTHALMOLOGY	2021/3	6.33	35
AI-Model for Identifying Pathologic Myopia Based on Deep Learning Algorithms of Myopic Maculopathy Classification and “Plus” Lesion Detection in Fundus Images	Han, W	FRONTIERS IN CELL AND DEVELOPMENTAL BIOLOGY	2021/10	7	31

### Analysis of top productive countries and their collaboration networks

**Figure 3:**
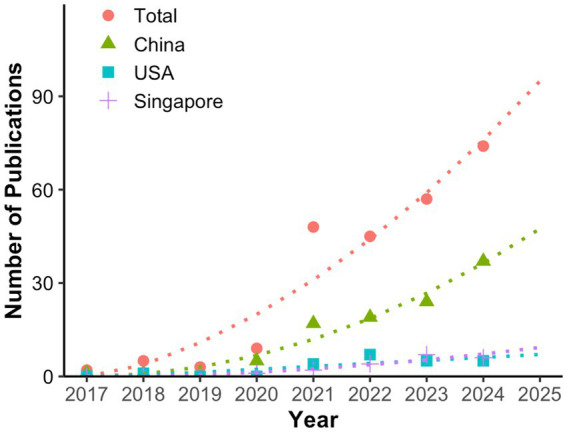
Publication trends and prediction curve of global and leading countries in AI-related HM research. This figure uses full counting, meaning each country listed in a paper contributes a weight of 1.

A total of 29 countries contributed to the research on this topic. Mainland China led with 111 publications (66.5%), followed by the United States with 20 papers (12.0%) and Singapore with 18 papers (10.8%). Figure 3 showed the publication trends of AI-related HM research in the top 3 productive countries. It highlighted that China experienced the fastest increase in the number of publications since 2020. This trend indicated that China was likely to maintain its leadership in this field, with a continued and steady rise in publication numbers.

**Figure 4:**
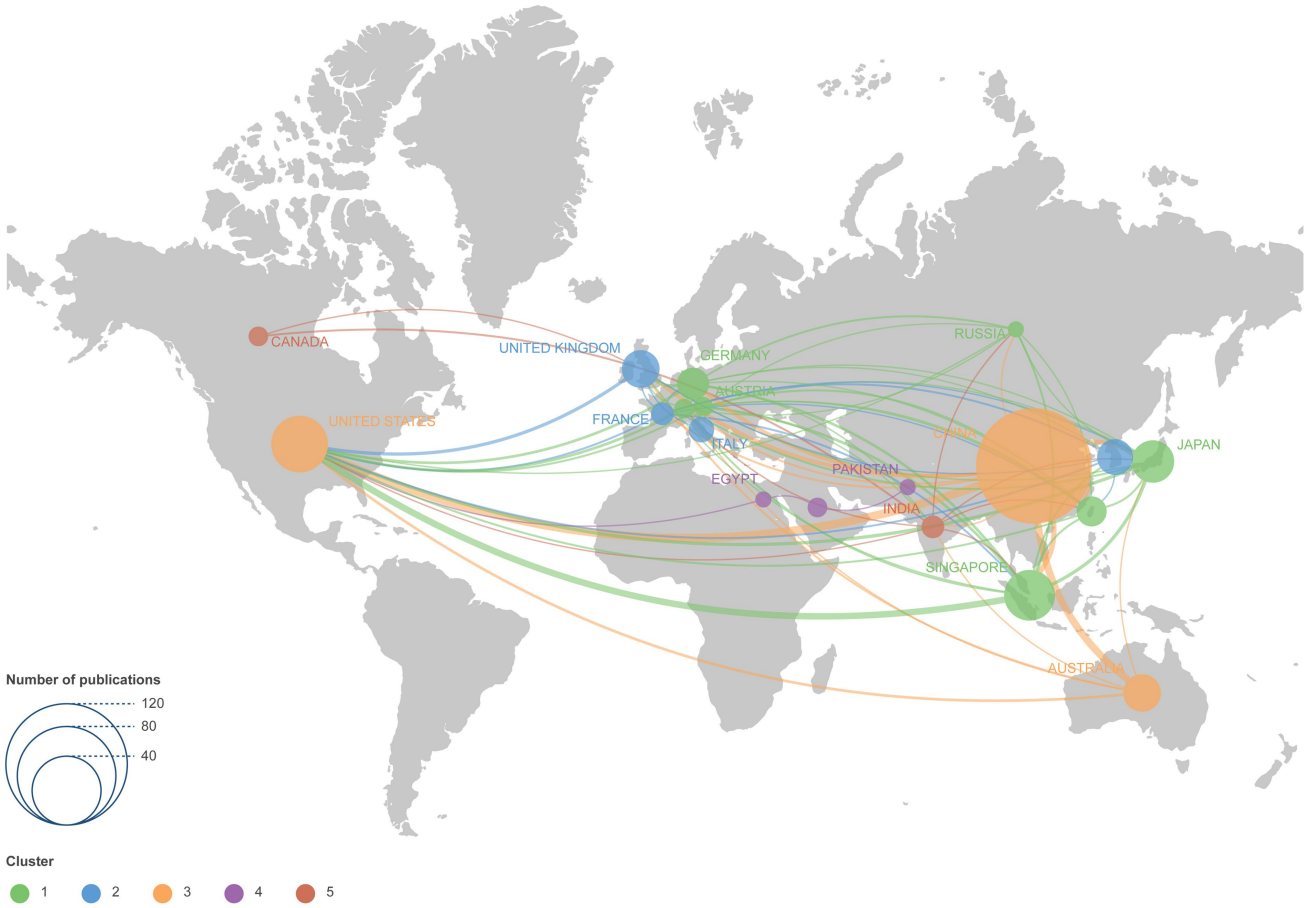
Co-authorship network visualization map of countries and regions. Each node represents a country or region, with the circle size reflecting the number of publications. Connecting lines represent collaboration between countries and regions.

Co-occurrence analysis of countries was also performed, revealing five distinct clusters (Figure 4): (1) Mainland China, the USA, and Australia; (2) the UK, Italy, South Korea, and France; (3) Austria, Germany, Japan, Russia, Singapore, Switzerland, and Taiwan China; (4) Saudi Arabia, Pakistan, and Egypt; (5) Canada and India. Countries with limited international collaboration were not represented in the network.

### Analysis of top productive institutions and their collaboration networks

**Figure 5:**
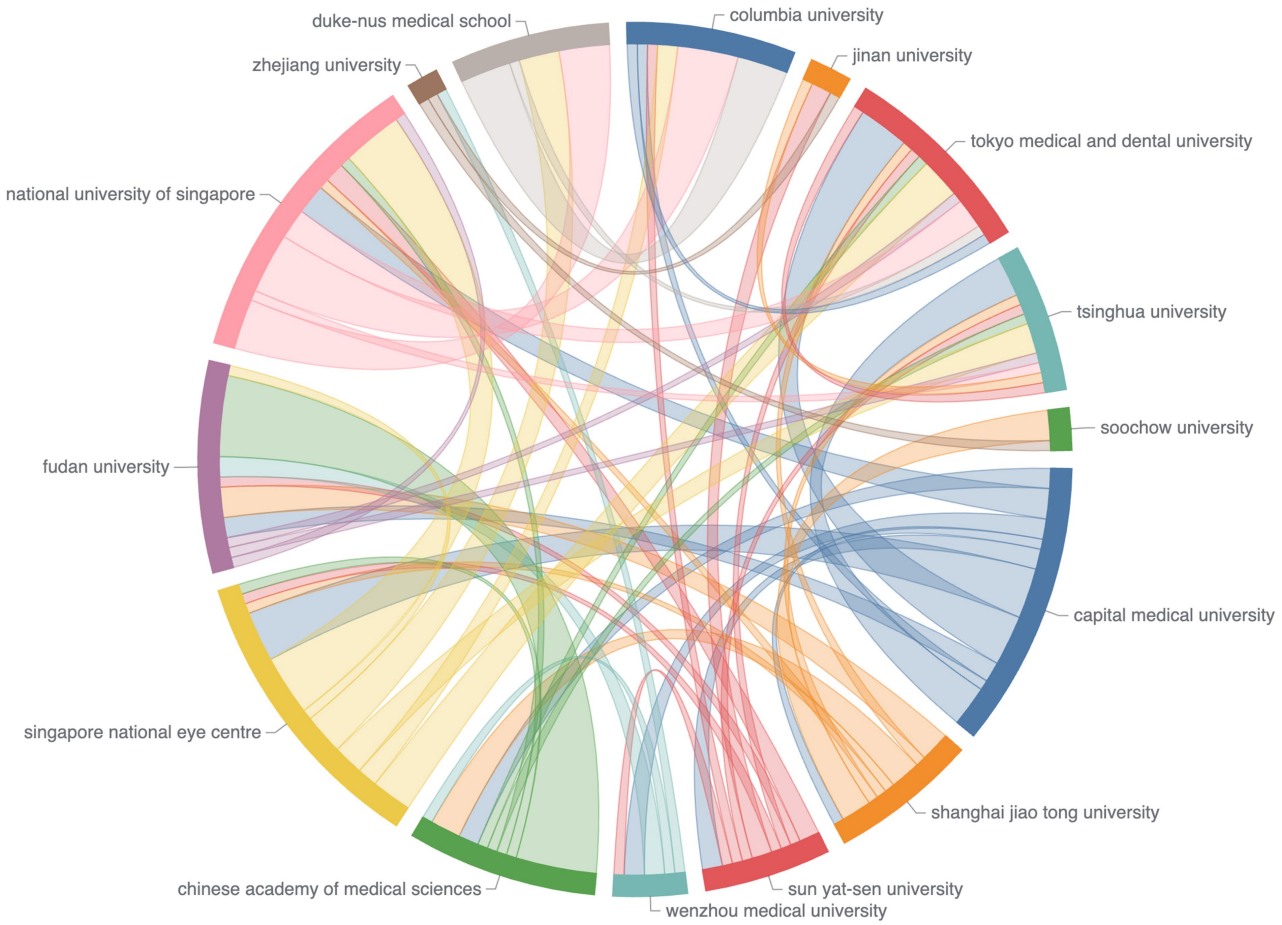
Chord diagram of co-authorship among the top institutions. Node data are arranged radially along the circumference, with weighted arcs (indicating collaboration strength) connecting the nodes.

A total of 318 institutions participated in the research. Notably, Capital Medical University (22, 13.2%), National University of Singapore (16, 9.6%), Singapore National Eye Center (16, 9.6%), and Shanghai Jiao Tong University (16, 9.6%) made substantial contributions. Supplementary Figure 1 presented the co-authorship network of institutions. While there was frequent collaboration within institutions, institutional collaborations across different countries remained less cohesive. Figure 5 offered a closer look at the cooperation among the top productive institutions.

### The leading journals, and authors

The journals publishing AI-related HM papers were quite diverse (Table 2). The journal Investigative Ophthalmology and Visual Science (IOVS) published the highest number of papers (12, 7.2%), followed by Translational Vision Science and Technology (TVST) and Frontiers in Medicine, with 11 (6.6%) and 6 (3.6%) publications, respectively. Among the top 10 journals, 8 were ranked Q1 in JCR (2023). Nearly half of the AI-related HM papers were published in ophthalmology journals (Table 3).

**Table 2:** Top 10 productive journals ranked by publication count.

Source title	Record count (%)	Citations	H-index	IF (2023)	JCR (2023)
Investigative Ophthalmology and Visual Science	12 (7.19)	51	4	5.0	Q1
Translational Vision Science and Technology	11 (6.59)	124	6	2.6	Q2
Frontiers in Medicine	6 (3.59)	37	4	3.1	Q1
Scientific Reports	6 (3.59)	80	4	3.8	Q1
Eye	5 (2.99)	39	3	2.8	Q1
Journal of Translational Medicine	4 (2.40)	17	3	6.1	Q1
Frontiers in Cell and Developmental Biology	4 (2.40)	54	4	4.6	Q1
American Journal of Ophthalmology	4 (2.40)	94	2	4.1	Q1
Eye and Vision	4 (2.40)	25	2	4.1	Q1
International Journal of Ophthalmology	4 (2.40)	8	3	1.9	Q2
BMC Ophthalmology	4 (2.40)	7	2	1.7	Q3

**Table 3:** Top 10 Web of Science categories of journals on AI-related HM research.

Field: Web of Science categories	Record count (%)
Ophthalmology	71 (42.52)
Computer Science	26 (15.57)
Engineering	19 (11.38)
General Internal Medicine	18 (10.78)
Research Experimental Medicine	12 (7.19)
Radiology Nuclear Medicine Medical Imaging	11 (6.59)
Science Technology Other Topics	11 (6.59)
Medical Informatics	6 (3.59)
Imaging Science Photographic Technology	5 (2.99)
Mathematical Computational Biology	5 (2.99)
Neurosciences Neurology	5 (2.99)

The top 10 authors in this field were listed in Table 4 according to the number of publications. The leading contributor in this field was Yang Weihua from Shenzhen Eye Hospital, China, with 10 publications (5.99%) and 84 citations. Closely following was Daniel Ting from the Singapore National Eye Centre, who published 8 papers (5.06%) and received 190 citations in total. Asian authors made up the majority of the top 10 authors list. The cooperation between authors was illustrated in Supplementary Figure 2, which was generated based on the Author Contribution Index to minimize potential bias caused by differences in author order (34). Although collaboration among authors was generally limited, it was clear that authors from the same country tended to collaborate more closely with one another.

**Table 4:** Top 10 authors ranked by publication count.

Author	Country	Latest affiliation	Publications (%)	Citations	Citations per item
Yang, Weihua	China	Shenzhen Eye Hospital	10 (5.99)	84	8.4
Ting, Daniel	Singapore	Singapore National Eye Centre	8 (5.06)	190	23.75
Ohno-Matsui, Kyoko	Japan	Tokyo Medical and Dental University	7 (4.43)	181	25.86
Wong, Tien Yin	Singapore	Singapore National Eye Centre	7 (4.43)	185	26.43
Jonas, Jost B.	Germany	Heidelberg University	7 (4.43)	107	15.29
Chen, Qiuying	China	Shanghai Jiao Tong University	7 (4.43)	41	5.86
Xu, Xun	China	Shanghai Jiao Tong University	7 (4.43)	36	5.14
Saw, Seang-Mei	Singapore	Singapore National Eye Centre	6 (3.80)	190	31.67
Hoang, Quan V.	Singapore	Singapore National Eye Centre	6 (3.80)	139	23.17
Marcus Ang	Singapore	Singapore National Eye Centre	6 (3.80)	133	22.17

**Figure 6:**
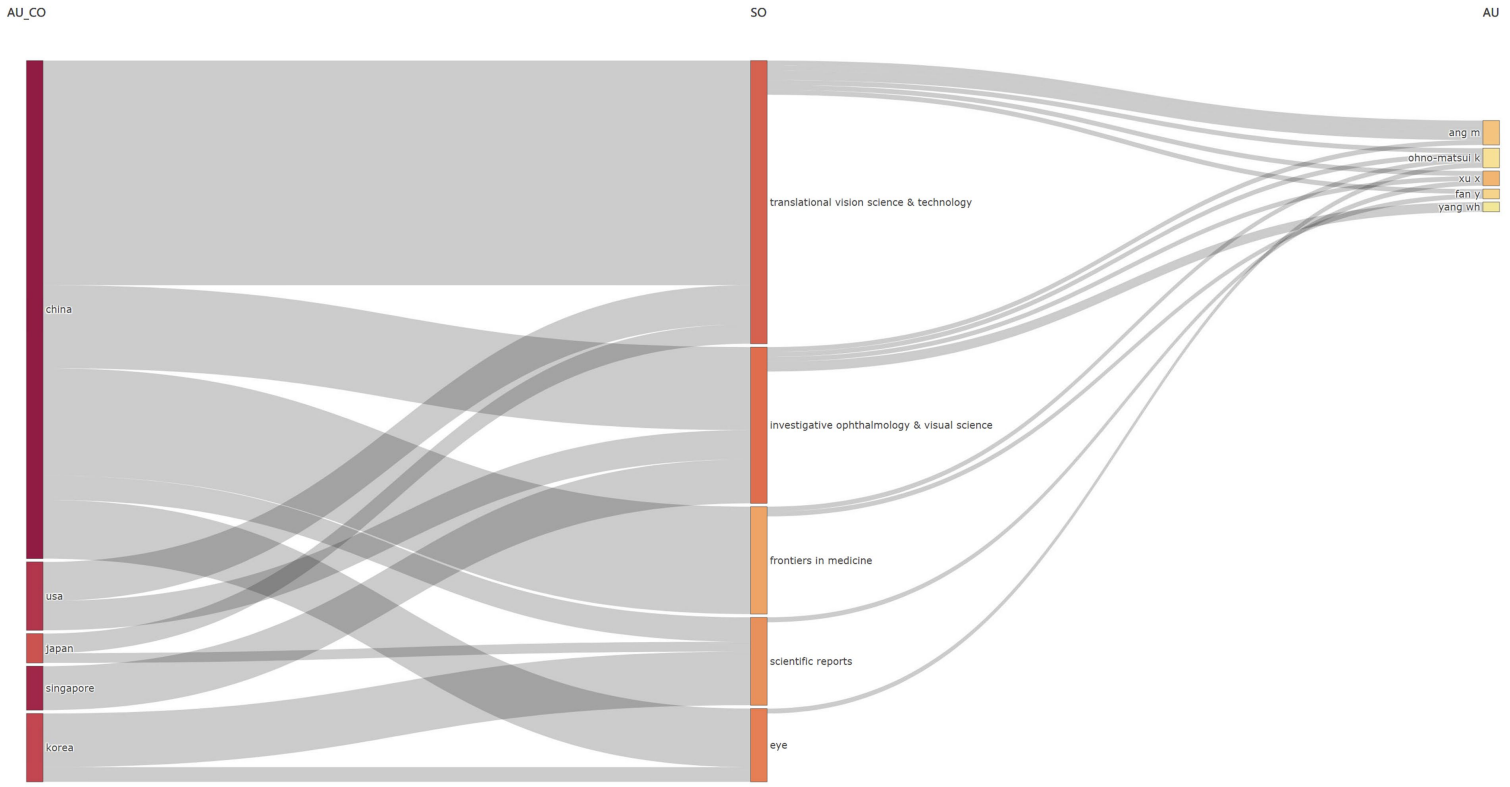
Three-field plot analysis displaying the journal preferences of authors from different countries. The three fields represent (1) AU_CO: Country, (2) SO: Source Journal, and (3) AU: Authors. The width of the nodes indicates the number of publications, while the width of the connecting lines reflects the level of collaboration.

We further analyzed the relationships among the top five countries, journals, and authors, and visualized the results with a three-field plot (Figure 6). This plot revealed the journal preferences among different countries and authors.

### Research hotspots

Keyword analysis identified the most frequently used terms and their connections within the field of AI-related HM research. Among 489 automatically recognized keywords we focused on those that appeared more than five times in the included publications. After merging duplicates and excluding irrelevant terms 40 keywords were identified. These were categorized into four primary clusters based on their co-occurrence frequencies (Supplementary Figure 3): an AI-focused cluster (red) an epidemiology-related cluster (green) an anatomy-related cluster (yellow) and a cluster associated with HM-related diseases (blue).

**Figure 7:**
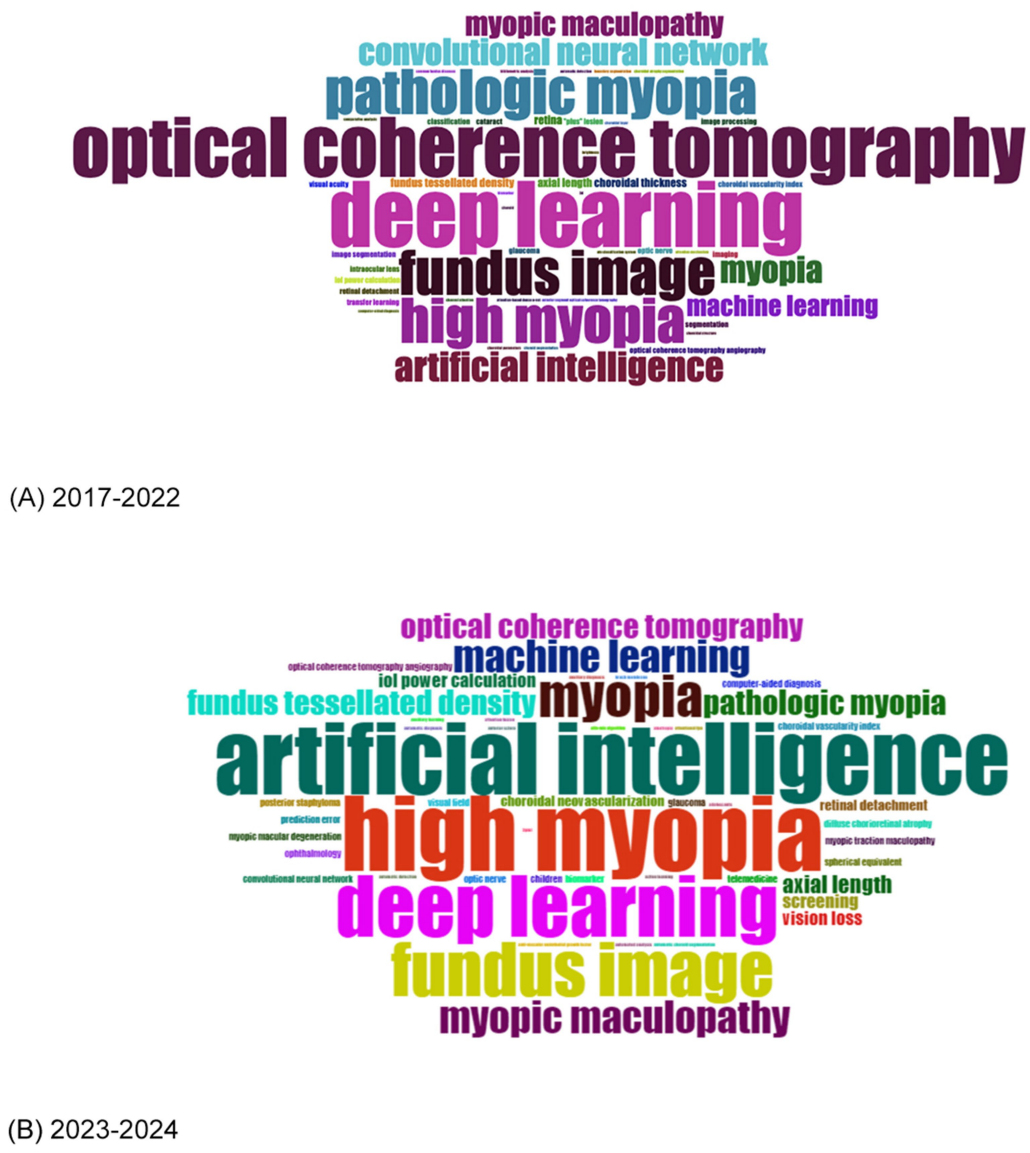
**(A)** Word cloud of the most frequent keywords from 2017 to 2022. **(B)** Word cloud of the most frequent keywords from 2023 to 2024.

To illustrate the most frequently used keywords, we created word clouds for two time periods: 2017–2022 (Figure 7A) and 2023–2024 (Figure 7B). “High myopia” and “artificial intelligence” were the most dominant keywords throughout the entire period, followed by “deep learning” and “fundus image.” Before 2022, key topics also included “pathologic myopia,” “optical coherence tomography (OCT),” “convolutional neural network,” and “myopic maculopathy.” After 2022, “myopic maculopathy” gained increased attention, while new keywords such as “screening,” “optical coherence tomography angiography (OCTA),” “fundus tessellated density” and “intraocular lens (IOL) power calculation” emerged. We also extracted the most common keywords to generate a trend topics plot using the bibliometrix package in R (Supplementary Figure 4).

**Figure 8:**
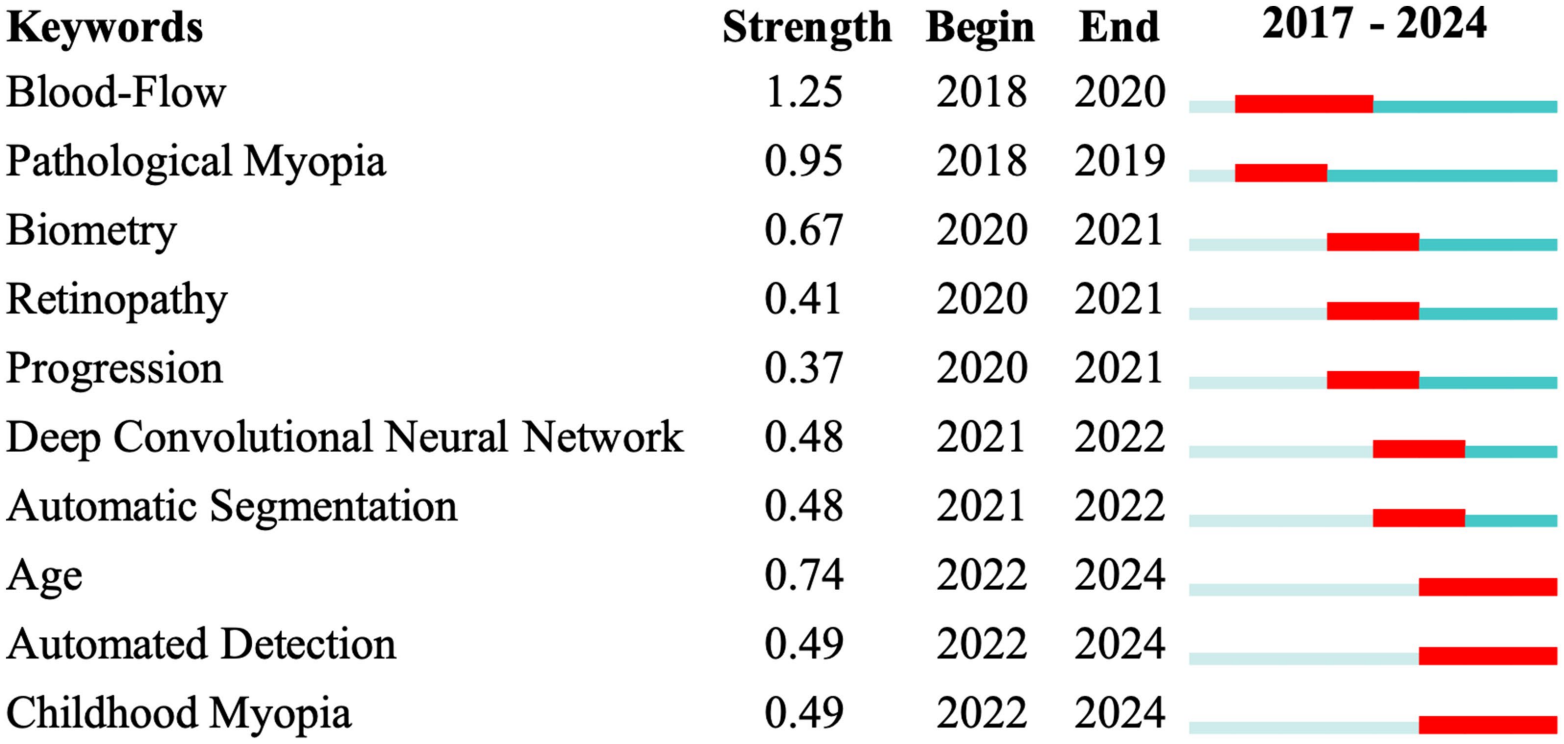
Burst map of Title-Abstract-Keywords. The numerical columns display the relative burst strength, start, and end times. In the line graph, the red portion represents the burst duration for each time series.

To further understand when these research hotspots emerged and how they evolved, Figure 8 was generated to display the burst strength of Title-Abstract-Keywords (TS) across different periods. The findings indicated three distinct research focus periods: before 2020 (blood flow and pathological myopia), 2020–2022 (biometry, retinopathy, progression, deep convolutional neural network and automatic segmentation), and 2022–2024 (automated detection, age and childhood myopia). These findings aligned generally with the trends identified in the trend topic results above.

## Discussion

This study explored AI research in HM by analyzing the trend of publications, publishing patterns, research activity characteristics, and emerging research hotspots through bibliometric data.

The rise in scientific publications and citations often reflects advancements in a specific research area. Our analysis indicated that the first AI-related HM paper was published in 2017, with low publication activity over the next few years (fewer than 10 papers per year). However, a significant increase occurred in 2021 (a 300% rise), followed by steady annual growth. According to our prediction model, this upward trend was expected to continue, reaching a plateau around 2028. This growth could be attributed to the rapid advancement of AI technologies and the increasing interest in their application to ophthalmology (10, 16). Additionally, the rise may also be linked to the recent global rise in HM incidence and the growing attention to HM and its complications (2, 35). Since 2022, publication levels remained consistently high (over 30 papers annually), potentially influenced by the release of ChatGPT in that year, which sparked broader interest in AI technologies and fueled the growth of “AI+” academic research (36, 37), including AI-powered disease detection (38), personalized treatment strategies (39), and drug development (39).

The most influential paper in this field was published by Liu YZ and his team in 2018 (33). It marked the first application of machine learning in China to analyze real-world data and develop an algorithm for predicting the onset of HM in school-aged children. The study’s high citation rate was largely due to its practical impact, as it demonstrated how big data and machine learning could enhance the prediction of HM outcomes using large-scale electronic health records. Notably, 7 of the top 10 most influential papers in this area involved AI applications for analyzing retinal images related to HM (40–46). This further suggests that fundus-related research is a key focus in the application of AI to the field of HM.

The volume of publications from a country or region often reflects its interest and expertise in a particular research area. In the field of AI-related HM studies, China led significantly (111, 66.5%), according to statistics from the WoS intrinsic toolkits, and was expected to maintain its dominance. This can be attributed to the high prevalence of HM in East Asian countries like China (47, 48), along with China’s recent national policies and funding initiatives focused on myopia (49, 50). Additionally, the rise of innovative Chinese companies specializing in AI-based fundus image analysis provided advanced tools and technologies (51, 52), further fueling research growth. These factors highlight how external support, alongside disease prevalence, plays a critical role in driving research progress.

International collaboration has become a preferred approach among researchers. China demonstrated the strongest global partnerships (total link strength of 69), while other countries also engaged in substantial collaborative efforts. This trend was probably driven by HM’s status as a global public health challenge (53), making joint research an essential strategy. However, AI-related HM studies remained concentrated in North America, Europe, Australia, and East Asia, with limited contributions from less developed regions, despite the significant prevalence of HM in some of these areas. This disparity underscores the need to expand research efforts in underserved regions. The application of AI, telemedicine, and LLMs might improve diagnosis, screening, and routine examinations for HM in these areas, bridging the gap in global research and healthcare equity (54, 55).

Academic collaboration among institutions mirrors the trend of international partnerships, showing significant activity. Capital Medical University stood out with the highest collaboration network (total link strength of 96) and served as the largest initiator of cooperative efforts, despite none of the top 10 authors being affiliated with it. Among the top 10 authors, nine were based in East Asia. Yang Weihua from Shenzhen Eye Hospital contributed the most publications (9 in English, 1 in Chinese), while Daniel Ting from the Singapore National Eye Centre received the highest total citations (190), averaging 23.75 citations per paper. This highlights the prominent role of East Asian researchers in advancing AI-related HM research.

The majority of AI-related HM articles were published in ophthalmology journals (71, 42.5%), with a notable portion also appearing in computer science journals (26/167). Among these, IOVS and TVST, both published by the Association for Research in Vision and Ophthalmology, led in publication volume and citation count, respectively. TVST was particularly popular among authors from China and the United States, while researchers from Singapore showed a preference for Investigative Ophthalmology & Visual Science. Most studies in this field primarily focused on clinical research, whereas the application of AI to animal models in HM remained limited. Notably, KhalafAllah et al. (56) utilized DLMs to explore the significant thinning of peripapillary tissues during the progression of HM in juvenile tree shrews. As reported, AI holds great promise in basic experimental research on HM—for instance, it can be integrated with gene-edited animal models and advanced imaging techniques to enhance the precision, efficiency, and scalability of such studies (57, 58). In fact, AI has already demonstrated remarkable success in basic research across other biomedical fields (59–61), yet its application in HM-related animal studies remains underexplored, highlighting the need for further investigation.

Thematic analysis of keywords helps clarify current research priorities and emerging trends. Based on VoS clustering and manual review, we categorized the included literature into several key application areas: epidemiology and screening (43, 46, 62), automated diagnosis (42, 45), disease monitoring and progression prediction (33, 63, 64), treatment planning (65–67), retinal image-based quantification (68–70), and classification and subtyping (44, 71). In parallel, a systematic breakdown of AI task categories revealed the frequent use of classification (44, 71, 72), segmentation (44, 69), prediction (33, 64, 65), quantification (68, 69), and multi-task learning (70, 73). These task types were often built on fundus imaging data, which remains central because of its diagnostic value, non-invasive nature, and wide accessibility (74, 75). Co-occurrence analysis further confirmed that HM-related retinopathy remains a consistently prominent research focus, likely due to its significant health burden and its status as a leading cause of best-corrected visual acuity loss worldwide (76, 77). Furthermore, the reliance on fundus image data for the diagnosis and treatment of HM-related retinopathy supported AI-driven analysis (78–80), facilitating the development of DLMs for improved diagnosis and classification (71). Epidemiology-related keywords, such as “screening” and “epidemiology” also gained prominence, likely in response to recent public health initiatives (49). The growing availability of real-world clinical data further strengthened AI models by providing rich external training sets (33, 42).

To better illustrate the evolving focus of research in this field, we divided the analysis into two periods: 2017–2022 and 2023–2024. This division was based on publication trends (with roughly equal publication volumes before and after 2022) and the significant AI event (36) (in December 2022, the release of ChatGPT-3.5 sparked global discussions on AI). In the first period (2017–2022), the word cloud prominently featured OCT. This can be attributed to its widespread use in ophthalmology in this period and its unique capability to capture high-quality fundus images (81–83). Researchers such as Sogawa et al. (45) and Ye et al. (62) aimed to develop DLMs to identify retinopathy in HM patients using OCT images, with AUC values exceeding 0.95. Liu et al. (69) and Wang et al. (72) also created AI models using OCT images to analyze choroidal parameters in HM cases. Notably, Yoo et al. (84) introduced a DLM to predict uncorrected refractive errors from posterior segment OCT images, further showcasing the potential of OCT-based AI applications in this field. In the second period (2023–2024), keywords became more varied, with no single term taking a clear lead. However, emerging topics like IOL implantation power calculation and screening suggested an increasing interest in applying AI to HM patients of different age groups. This included AI-assisted IOL implantation for age-related cataracts in the elderly (65–67, 85) and AI models supporting HM screening in school-aged children (63, 68, 86–88). Additionally, other emerging keywords, such as OCTA, highlighted the progression of retinal imaging techniques for HM, from basic fundus photography to OCT, wide-field OCT, and OCTA (83). This trend underscores the increasing sophistication and diversity of diagnostic technologies in this field.

Our analysis of TS burst trends aligned with the previous findings. Before 2022, research primarily focused on fundus-related topics in HM. Among the key terms, “blood flow” emerged as an early and enduring hotspot. For instance, Zhou et al. (89) employed OCT with a split-spectrum amplitude-decorrelation angiography algorithm to evaluate parameters, such as the foveal avascular zone area and macular blood flow, in HM patients. After 2022, the focus shifted toward “age,” with an emphasis on pediatric HM populations. Zhao et al. (86), for example, developed an AI-based fully automated system for analyzing retinal vascular morphology in children with HM. This shift might be driven by an evolving understanding of HM-related diseases, with a growing emphasis on early diagnosis and intervention before complications arise. At the same time, there was increasing awareness of the need to integrate HM management into long-term care strategies across all age groups. For instance, Wang et al. (64) developed models to accurately predict long-term visual acuity in HM eyes based on clinical and imaging data. These evolving trends highlight the expanding and increasingly specialized applications of AI in HM research.

Despite the promising potential of AI in HM, several challenges persist. First, many studies using DLMs are confined to their own datasets and have not been widely applied or validated in real-world clinical settings. This gap may be attributed to multiple factors, including data privacy concerns, regulatory barriers, limited clinician acceptance, and the inconsistent quality of real-world data—particularly imaging. For example, in Zhao et al.’s pediatric HM screening model (86), although the reported accuracy reached 94.19%, its strict requirements for image quality significantly limited its applicability in routine ophthalmic practice, where such ideal conditions are not always met. Second, as previously mentioned, most AI applications in HM focus on clinical research, with a notable lack of foundational studies. This is likely due to the lack of animal models that can accurately replicate the pathological features of HM in human eyes, thus limiting AI’s potential. Additionally, the absence of large-scale public datasets of HM hinders the development of models and algorithms. We advocate for open-source data sharing to accelerate progress in this field. This could include forming international consortiums for multi-center data collection and establishing standardized, ethical frameworks for secure data sharing (90). Finally, the application of AI in HM also raises ethical concerns, such as the lack of transparency in how AI models make clinical decisions, which may reduce clinician trust and patient acceptance (91, 92). Incorporating interpretable AI models and clear reporting of decision-making processes can help improve transparency and support ethical integration into clinical practice (93).

Several key areas remain to be explored in the future. First, there is an urgent need to develop DLMs for multimodal imaging of HM, which would enable more precise and comprehensive diagnosis and treatment (70, 73). Currently, this remains an underexplored area. Second, improving the accessibility of these algorithmic models is crucial. HM, as a chronic condition requiring long-term follow-up (94), often coexists with other eye diseases, including glaucoma (9). This increases the demand for healthcare resources, which are unevenly distributed across the globe (95, 96). Barriers such as poor internet connectivity, limited computing infrastructure, and a lack of trained personnel hinder AI adoption in low-income regions. Developing low-cost, efficient models could help bridge this gap and support wider clinical use. Lastly, there is a growing demand for an integrated model that can diagnose, classify, guide treatment, and predict postoperative outcomes, addressing the full spectrum of clinical needs rather than focusing solely on one aspect of HM.

This study represented the first bibliometric analysis of AI-related research in HM, particularly in the context of the global rise in AI interest following the advent of technologies like ChatGPT. Our findings will offer valuable insights into the evolution of this field, helping researchers identify key trends and focus areas for future investigations. However, the study does have some limitations. First, although we included two major medical databases—WoSCC and MEDLINE—and added CSCD to reflect China’s leading role in the field, some non-English databases were still excluded. Among them, only WoSCC provided comprehensive bibliometric data, while others may lack complete publication statistics. Additionally, differences in author positions may introduce bias in evaluating individual influence and collaboration networks. As there is currently no universally accepted method to quantify author contributions by position, we referred to the percentage-based Author Contribution Index to construct the author collaboration network (Supplementary Figure 2). Furthermore, when calculating publication counts for countries using the WoS built-in toolkit, internationally co-authored articles were counted for all listed countries, as the tool only supports full counting. This resulted in an inflated representation of each country’s publication count. The same approach was also applied to the analysis of institutions and authors. To provide a more accurate representation of relative proportions, the global total publication count in Figure 3 was calculated by summing the publication counts of each country.

## Conclusion

This study provided an overview of global research on AI in the field of HM for the first time. Recent advancements in AI applications for HM have been significant, and this trend is expected to continue. Future research will likely focus on multimodal imaging and improving algorithm availability.

## Data Availability

The original contributions presented in the study are included in the article/Supplementary material, further inquiries can be directed to the corresponding author.

## Supplementary material

Supplementary Figure 1 Co-authorship network visualization map of institutions. Each node represents an institution, with the circle size reflecting the number of publications. Connecting lines represent collaboration between institutions.

Supplementary Figure 2 Co-authorship network visualization map of authors. Each node represents an author, with the circle size reflecting the number of publications. Connecting lines represent collaboration between authors.

Supplementary Figure 3 Keyword co-occurrence map showing five clusters: an AI-focused cluster (red), an epidemiology-related cluster (green), an anatomy-related cluster (yellow), and a cluster related to HM-associated diseases (blue). Node size represents frequency, and connecting lines show co-occurrence between keywords.

Supplementary Figure 4 Trend topics of author’s keywords. The position of the circles on the timeline indicates the median year of high-frequency keywords, with circle size representing frequency. The blue segments illustrate the time range between the first and third quartile of keyword usage.
