# Supplementary figures and images for: Global trends and hotspots in artificial intelligence for high myopia: a bibliometric analysis
Source: Front Med (Lausanne). 2025 May 9;12:1567440. doi: 10.3389/fmed.2025.1567440 (PMC12098612; doi:10.3389/fmed.2025.1567440)

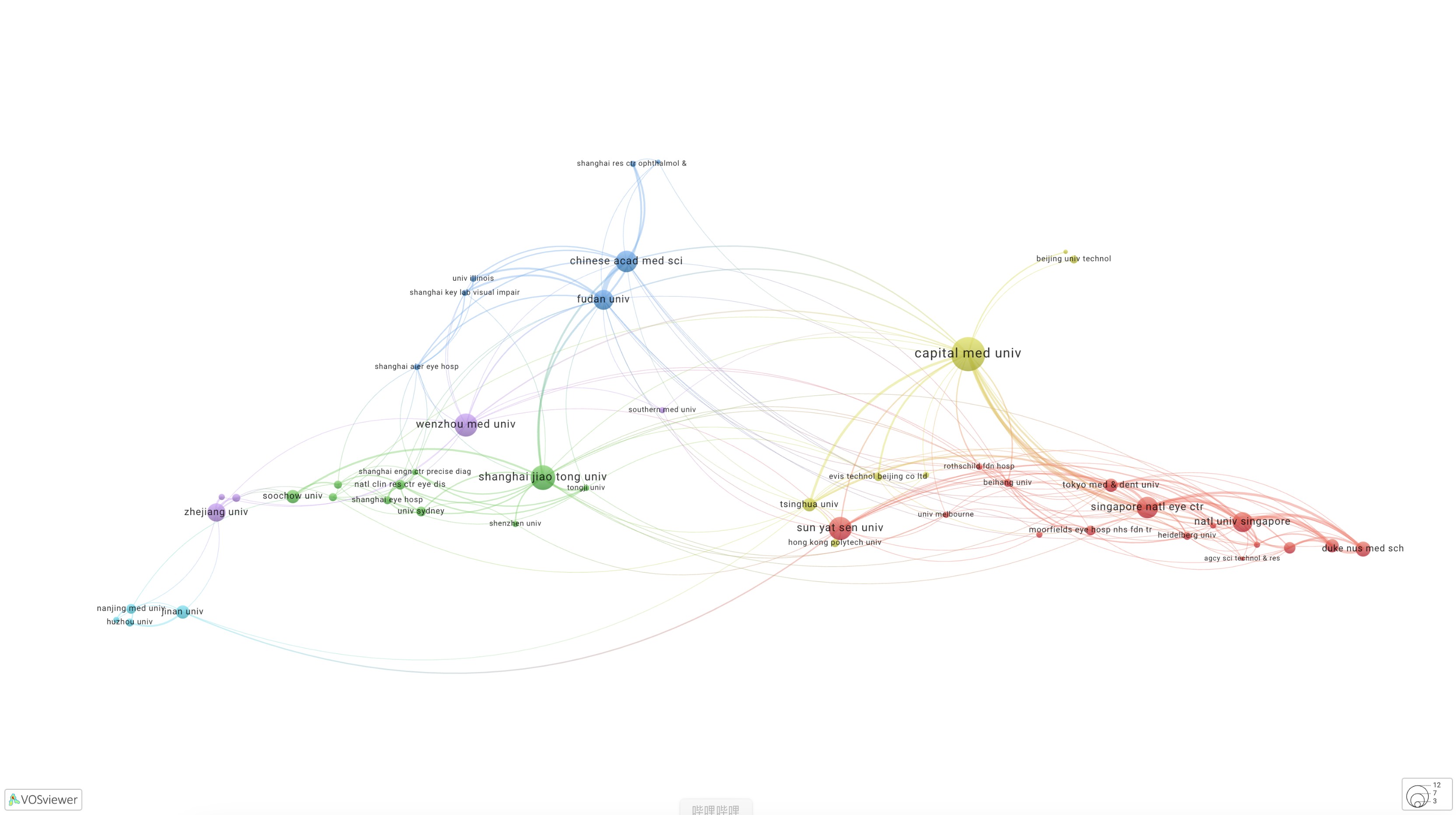

Supplement: Supplementary Figure 1 — Co-authorship network visualization map of institutions. Each node represents an institution, with the circle size reflecting the number of publications. Connecting lines represent collaboration between institutions. [file Image_1.JPEG]

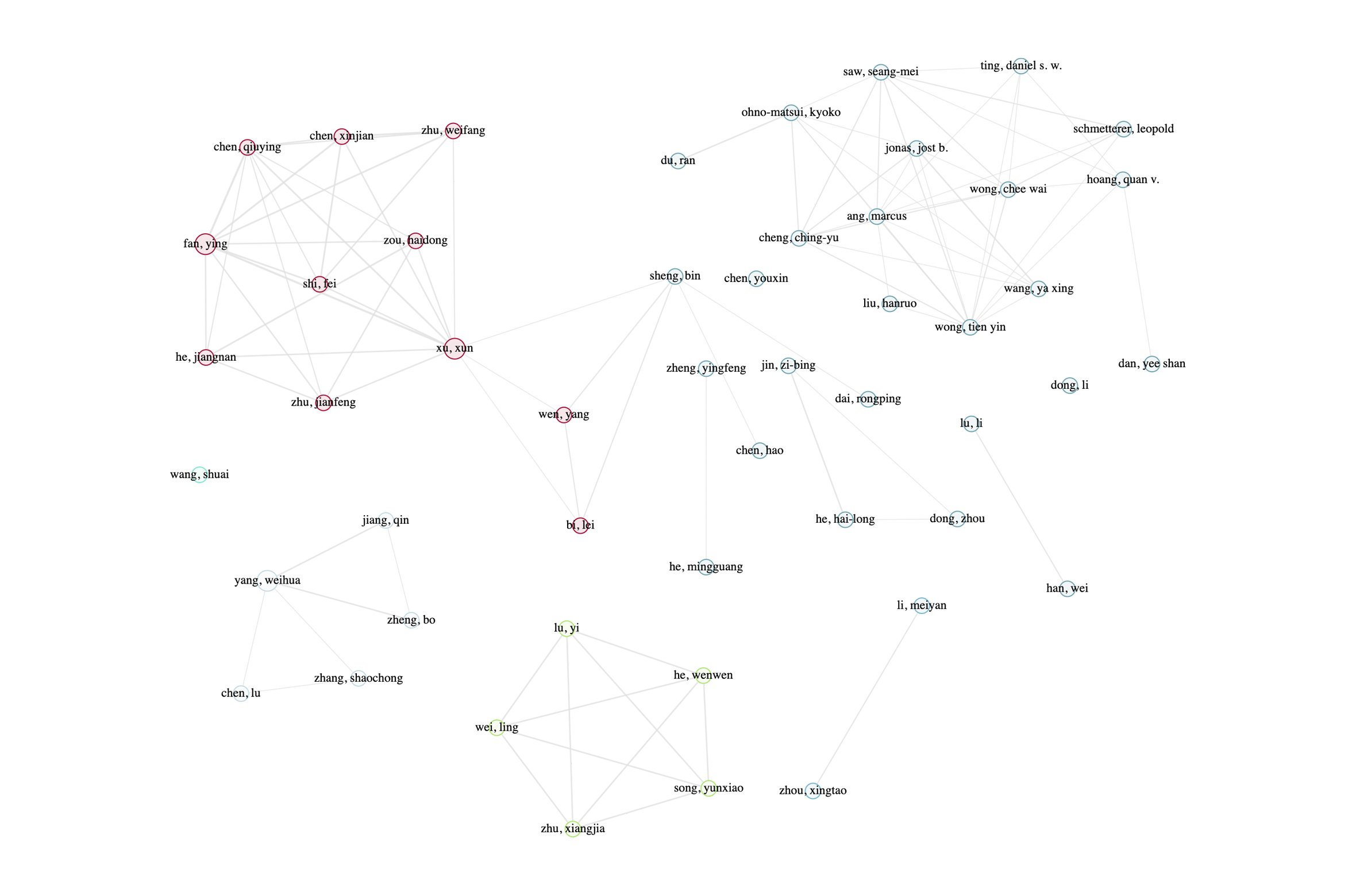

Supplement: Supplementary Figure 2 — Co-authorship network visualization map of authors. Each node represents an author, with the circle size reflecting the number of publications. Connecting lines represent collaboration between authors. [file Image_2.JPEG]

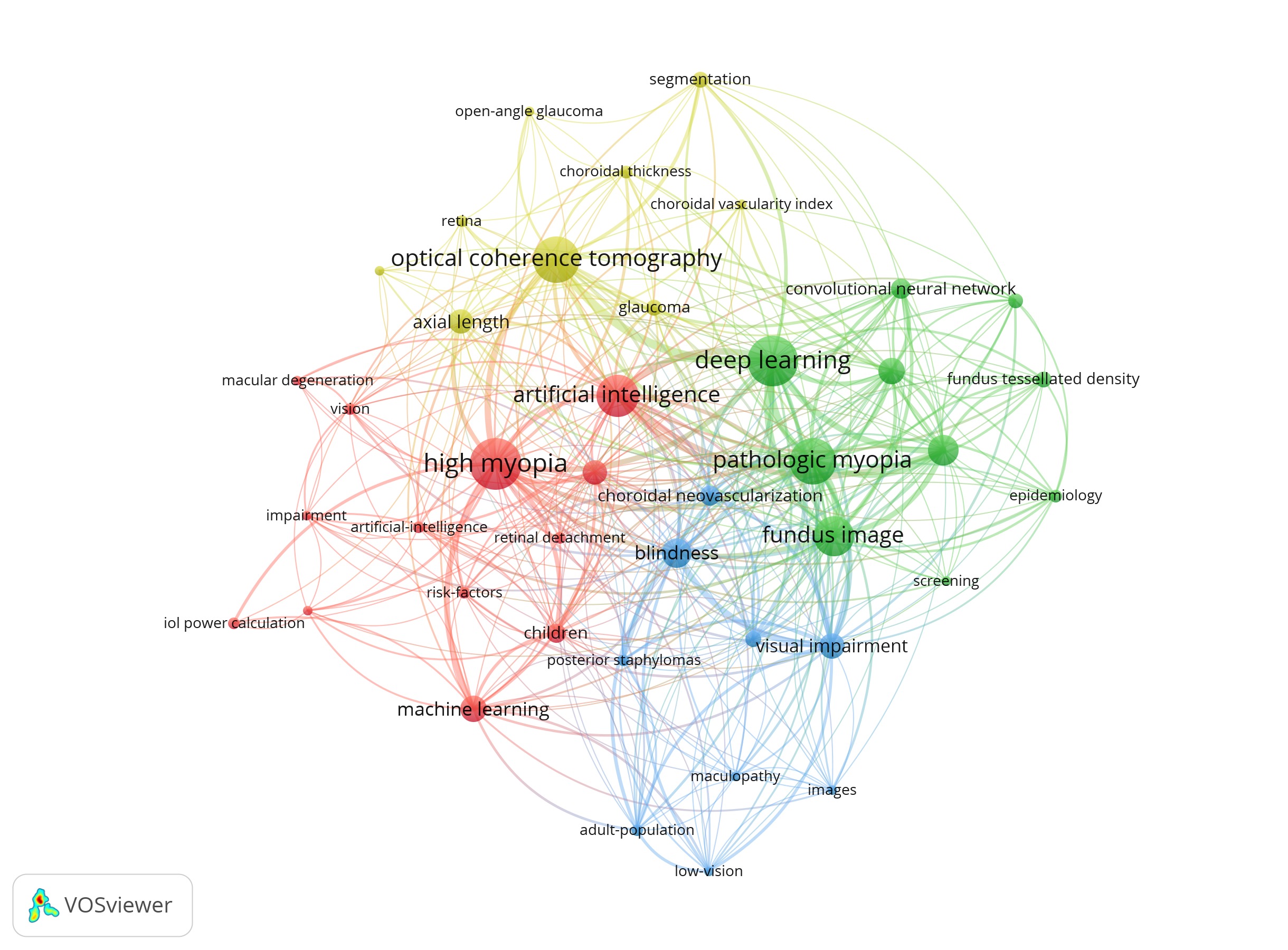

Supplement: Supplementary Figure 3 — Keyword co-occurrence map showing five clusters: an AI-focused cluster (red), an epidemiology-related cluster (green), an anatomy-related cluster (yellow), and a cluster related to HM-associated diseases (blue). Node size represents frequency, and connecting lines show co-occurrence between keywords. [file Image_3.JPEG]

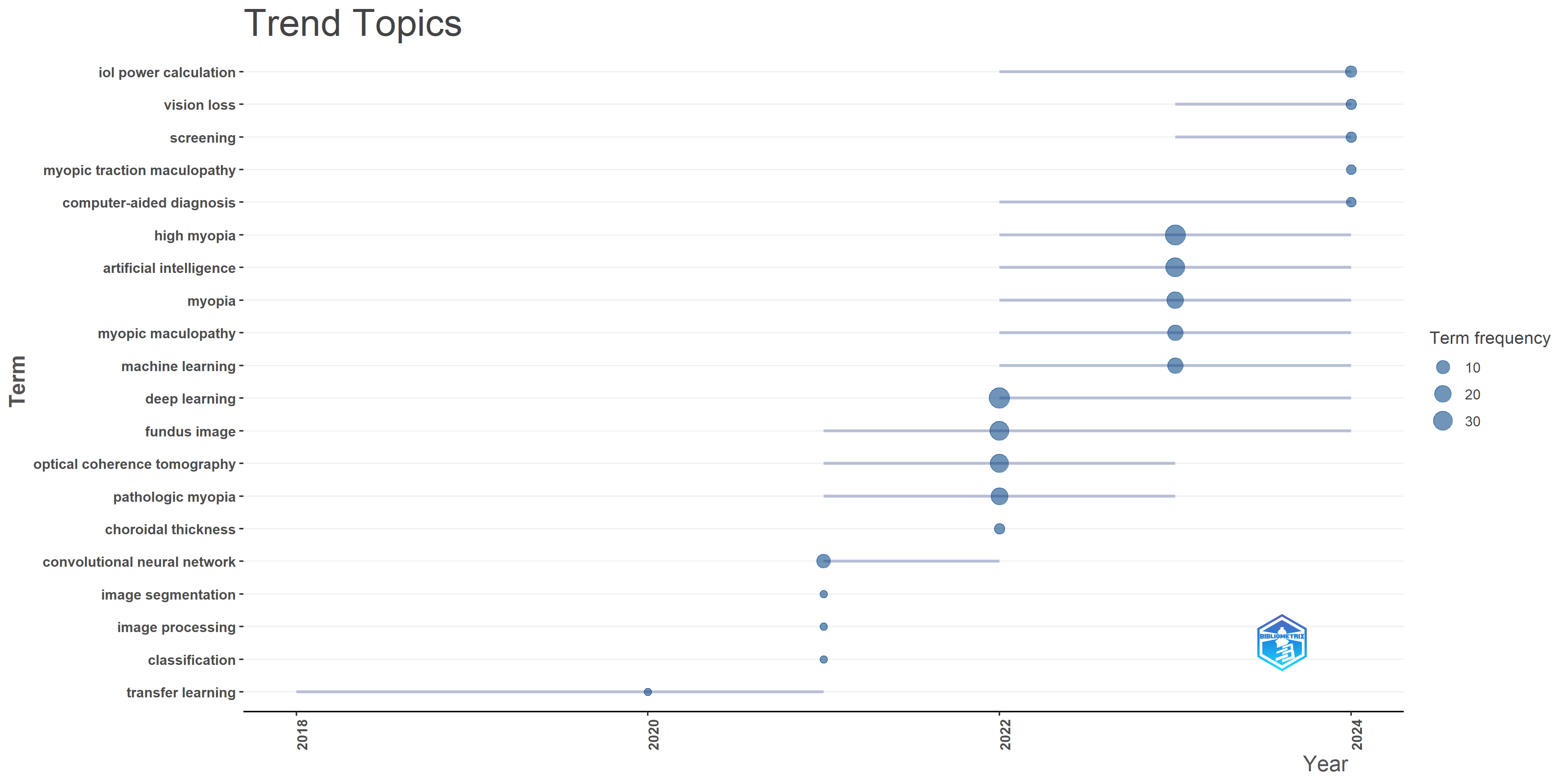

Supplement: Supplementary Figure 4 — Trend topics of author’s keywords. The position of the circles on the timeline indicates the median year of high-frequency keywords, with circle size representing frequency. The blue segments illustrate the time range between the first and third quartile of keyword usage. [file Image_4.JPEG]
